# Identification and characterization of a new pathologic mutation in a large Leber hereditary optic neuropathy pedigree

**DOI:** 10.1186/s13023-024-03165-2

**Published:** 2024-04-06

**Authors:** Sonia Emperador, Mouna Habbane, Ester López-Gallardo, Alejandro del Rio, Laura Llobet, Javier Mateo, Ana María Sanz-López, María José Fernández-García, Hortensia Sánchez-Tocino, Sol Benbunan-Ferreiro, María Calabuig-Goena, Carlos Narvaez-Palazón, Beatriz Fernández-Vega, Hector González-Iglesias, Roser Urreizti, Rafael Artuch, David Pacheu-Grau, Pilar Bayona-Bafaluy, Julio Montoya, Eduardo Ruiz-Pesini

**Affiliations:** 1 Departamento de Bioquímica, Biología Molecular y Celular, Universidad de Zaragoza, 50009- and 50013 Zaragoza, Spain; 2 Instituto de Investigación Sanitaria (IIS) de Aragón, 50009, Zaragoza, Spain; 3 Centro de Investigaciones Biomédicas en Red de Enfermedades Raras (CIBERER), Instituto de Salud Carlos III, 28029 Madrid, Spain; 4 Servicio de Oftalmología, Hospital Clínico Universitario Lozano Blesa, 50009 Zaragoza, Spain; 5 Servicio de Oftalmología, Hospital Universitario de Toledo, 45004 Toledo, Spain; 6 Servicio de Oftalmología. Hospital Universitario Río Hortega, 47012 Valladolid, Spain; 7 Instituto Oftalmológico Recoletas, 47004 Valladolid, Spain; 8 Instituto Oftalmológico Fernández-Vega, 33012-Oviedo, Asturias, Spain; 9 Instituto de Productos Lácteos de Asturias, Consejo Superior de Investigaciones Científicas (IPLA-CSIC), 33300-Villaviciosa, Asturias, Spain; 10 Departament de Bioquímica Clínica, Institut de Recerca Sant Joan de Déu, 08950 Barcelona, Spain; 11 Instituto de Biocomputación y Física de Sistemas Complejos (BIFI), Universidad de Zaragoza, 50018, Zaragoza, Spain; 12 Present Address: Laboratoire Biologie Et Santé, Faculté Des Sciences Ben M’Sick, Hassan II University of Casablanca, 20670 Casablanca, Morocco; 13 Present Address: Certest Biotec, 50840-San Mateo de Gállego, Zaragoza, Spain

**Keywords:** Leber hereditary optic neuropathy, Mitochondrial DNA, Pathologic mutation, Large pedigree, Incomplete penetrance

## Abstract

**Background:**

Most patients suffering from Leber hereditary optic neuropathy carry one of the three classic pathologic mutations, but not all individuals with these genetic alterations develop the disease. There are different risk factors that modify the penetrance of these mutations. The remaining patients carry one of a set of very rare genetic variants and, it appears that, some of the risk factors that modify the penetrance of the classical pathologic mutations may also affect the phenotype of these other rare mutations.

**Results:**

We describe a large family including 95 maternally related individuals, showing 30 patients with Leber hereditary optic neuropathy. The mutation responsible for the phenotype is a novel transition, m.3734A > G, in the mitochondrial gene encoding the ND1 subunit of respiratory complex I. Molecular-genetic, biochemical and cellular studies corroborate the pathogenicity of this genetic change.

**Conclusions:**

With the study of this family, we confirm that, also for this very rare mutation, sex and age are important factors modifying penetrance. Moreover, this pedigree offers an excellent opportunity to search for other genetic or environmental factors that additionally contribute to modify penetrance.

**Supplementary Information:**

The online version contains supplementary material available at 10.1186/s13023-024-03165-2.

## Background

Leber hereditary optic neuropathy (LHON) is an important cause of registrable blindness. Over 90% of patients worldwide harbor one of the three pathogenic mitochondrial deoxyribonucleic acid (mtDNA) mutations at positions m.3460G > A, m.11778G > A, and m.14484 T > C [1]. However, not all individuals carrying one of these mutations will develop the disease, highlighting the incomplete penetrance. Thus, studies of patients harboring these mutations primarily defined the penetrance-modifying factors associated with this disease, such as genetic causes like heteroplasmy or mitochondrial haplogroup, environmental influences like smoking, or physiological aspects like sex or age [2]. Most studies were based on pedigrees with a very limited number of maternally related individuals. However, a pedigree carrying the m.11778G > A mutation, including 129 maternally related individuals (32 affected), has been described [3].

The remaining 10% of LHON cases are due to a set of individually very rare mutations. It appears that the clinical features and some penetrance-modifying factors of these variants may be the same as those associated with the more common LHON mutations. However, a meta-analysis of pedigrees with these very rare mutations has not been performed, and no large pedigree carrying any of these very rare mutations has been reported.

We report a large LHON pedigree harboring a novel genetic variant in an mtDNA gene for a respiratory complex I (CI) subunit, perform studies in cytoplasmic hybrids (cybrids), and analyze the modifying factors for its incomplete penetrance.

## Materials and methods

### Pedigree Information

Institutional review boards of all participating centers approved this study (CEICA CP-05/2020). Informed consent was obtained from the subjects.

**Fig. 1:**
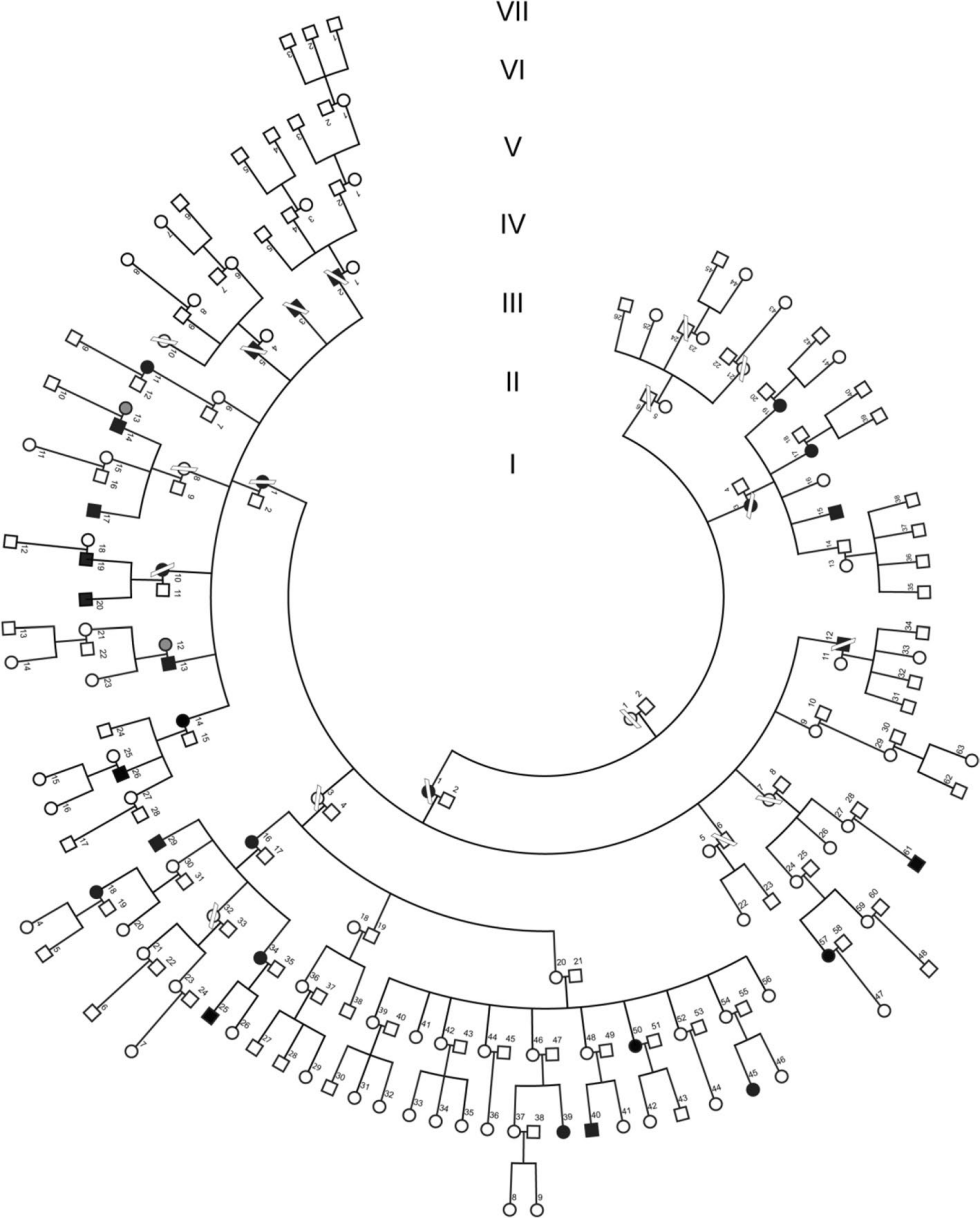
Seven-generation Leber hereditary optic neuropathy pedigree (LHON). Individuals affected by LHON are marked in black and those with blindness due to other causes are marked in gray

Patient 1 (VI-39) (Fig. 1): A 25-year-old female patient complained of painless subacute visual loss in her right eye (OD) in August 2018, which had lasted a few weeks. Her best-corrected visual acuity (BCVA) was 20/200 in her OD and 20/20 in her left eye (OS). Her optic discs appeared flat, with a small nasal ganglion cell layer (GCL) thinning and a slight temporal swelling of the retinal nerve fibre layer (RNFL) in OD (Figure S1). In this eye, the 30–2 visual field (VF) showed a small central scotoma, and a relative afferent pupillary defect was found. No family history of optic neuropathy was reported.

She was diagnosed with retrobulbar optic neuritis, and intravenous steroids treatment was initiated. Brain magnetic resonance imaging (MRI), lumbar puncture, self-immunity, coagulation, toxicology, and serology tests were normal. Since treatment did not improve the patient’s condition, and neurophysiological tests did not support a demyelinating etiology, neuromyelitis optica was suspected.

In November 2018, the patient suffered severe visual loss in her OD. The BCVA was finger counting (OD) and 20/20 (OS), although it worsened to hand movements (OD) and 20/32 (OS) a few days after she was admitted to the hospital. A big central scotoma was found in the VF of her OD, and a small central scotoma in her OS (Figure S2). The OD optic disc was pale, and OS showed peripapillary telangiectasia. There was slight RNFL bilateral thickening as well as a severe GCL thinning in her OD. LHON was highly suspected, genetic tests were performed, and 900 mg/day idebenone treatment was initiated.

In March 2019, BCVA had evolved to hand motion in both eyes. VF was nearly abolished bilaterally, and GCL were severely affected in both eyes (Figure S3). Although the patient completed 2 years of treatment with idebenone, visual function was not recovered.

Patient 2 (VI-40) (Fig. 1): A 20-year-old male, first cousin of case 1, went to the hospital in July 2020 with a painless visual loss in both eyes, over one month in OS and a week in OD. His BCVA was 20/200 in OD and hand movements in OS. Both optic discs were pale, but no telangiectasia was observed. A big central scotoma was found in his OS VF and a smaller one in his OD (Figure S4). RNFL was slightly thickened, while GCL showed a diffuse severe thinning in OS and thinning of the nasal sector in OD.

Although MRI and neurophysiology tests were requested, due to the previous findings and the family background of LHON, the treatment with 900 mg/day idebenone was started in September 2020. However, 2 months later, it had to be discontinued due to high transaminase levels. At that moment, BCVA was counting fingers in both eyes, VF were abolished, both optic discs were pale with temporal RNFL thinning, and GCL was severely affected bilaterally (Figure S5).

In June 2022, BCVA had evolved to hand movements, and there was severe RNFL thinning in both eyes (Figure S6). Transaminase levels went back to normal 6 months after stopping the treatment with idebenona, but this was not prescribed again.

Patient 3 (VI-45) (Fig. 1): A 15-year-old female, first cousin of patients 1 and 2, visited the emergency department in March 2022 due to progressive, painless visual loss in her OD lasting one month. BCVA was counting fingers (OD) and 20/20 (OS). Despite her family background, she was admitted to the Neurology department for the study of possible retrobulbar neuritis.

OD showed a wide central scotoma, mild thickening of RNFL, and severe thinning of GCL. In OS, only minimal thinning of GCL was observed.

Although treatment with 900 mg/day idebenone began in March 2022 and was well-tolerated, one month later, BCVA deteriorated to hand movements in OD and 20/200 in OS. Peripapillary telangiectasia was found in OS, along with bilateral RNFL thickening, drastic thinning of GCL in OD, abolition of OD visual field, and central scotoma in OS (Figure S7).

By September 2022, BCVA had decreased to hand movements in both eyes, with abolished VF, thinner and diffuse RNFL, and severe GCL thinning.

In February 2023, with the patient still under idebenona treatment, BCVA dropped to light perception in OD and hand movements in OS. Atrophy of both RNFL and GCL had progressed in both eyes.

Patient 4 (V-61) (Fig. 1): A 38-year-old man presented with bilateral vision loss, initially in OS in May 2022. He described rapid vision loss characterized by an inability to see straight ahead. The patient had undergone laparoscopic appendectomy in November 2021. No other associated risk factors were identified. Notably, the patient had a history of COVID in April 2022, one month before the onset of vision loss.

At the initial diagnosis, BCVA was 20/20 in OD and 20/200 in OS. Vision loss progressed over four months in both eyes, reaching a BCVA of light perception in September 2022. Cranial MRI revealed a small nonspecific lesion in the left hemicerebellar cortex and mild dilation of the ventricular system without an underlying cause.

Octopus visual field testing (30–2) showed a centrocecal campimetric defect in OS in June 2022, deepening in successive explorations until presenting absolute scotomas in both eyes in October 2022 (OD: MD 28.0 dB, MS: 2.8 dB; OS: MD 27.8 dB, MS 27.6 dB) (Figure S8).

Dilated fundus examination and optical coherence tomography (OCT) were within normal limits in August 2022. However, in September 2022, along with an afferent relative pupillary defect in OS, selective temporal atrophy was revealed in OS, with no telangiectasia (Figure S9).

OCT scans in October 2022 the patient showed RNFL thickness of 102 µm in OD and 104 µm in OS, and ganglion cell density of 74 µm in OD and 67 µm in OS. By March 2023, these values decreased to 80/70 µm and 56/53 µm respectively (Figure S10).

In addition, electroretinography (ERG) and visual evoked potential studies (VEP) in September 2022 showed a lack of reproducibility of bilateral P100, indicating bilateral optic nerve involvement without signs of lesion in cones and rods at the time.

Since August 2022, the patient has been under treatment with 900 mg/day of idebenone with poor response.

Patient 5 (V-57) (Fig. 1): A 48-year-old woman, a cousin of patient 4, sought consultation in August 2022 for acute vision loss in OD. Her BCVA at the time was light perception in OD and 20/25 in OS. Octopus visual field (30–2) examination revealed a large central scotoma in OD*.* Only one week later, the patient reported similar symptoms in OS, with a maximum BCVA of 20/63. She complained of a loss in the visual field in both eyes. Octopus visual field (30–2) demonstrated a minimal central island of vision in OD (MS 1.7 dB, MD 28.5 dB) and a large central scotoma in OS (MS 25.2 dB, MD 4.9 dB) (Figure S11).

Changes in dilated fundus examination began in September 2022, where temporal telangiectasias were appreciated in OS, and selective temporal atrophy was observed in OD (Figure S12). Atrophy was also reflected in the OCT scan, with RNFL thickness of 111 µm in OD and 94 µm in OS, and ganglion cell density of 70 µm in OD and 80 µm in OS in August 2022. By May 2023, these values decreased to 63/60 µm and 52/56 µm respectively (Figure S13).

A brain MRI identified 10 small lesions in the periventricular white matter and corpus callosum, that showed an isointense signal on T1, hyperintense on T2 and FLAIR, with no enhancement after contrast, indicative of multiple sclerosis (MS). No oligoclonal bands were detected, ruling out this diagnosis.

Neurophysiological study revealed severe optic nerve involvement, with no reproducibility of P100 in the right optic nerve and a slight delay in the left optic nerve.

Since October 2022, the patient has been under treatment with 900 mg/day of idebenone, with poor response.

Patient 6 (V-11) (Fig. 1): A 45-year-old female presented with bilateral painless loss of vision since 2017 (Figure S14). She was prescribed 900 mg/day idebenone until 2018 but discontinued the medication due to reported side effects. BCVA was 20/160 (OD) and 20/400 (OS). ERG showed normal scotopic and photopic responses. Multifocal ERG was normal in both eyes. VEP pattern was outside normal limits in both eyes.

Patient 7 (IV-14) (Fig. 1): A 71-year-old female manifested bilateral painless vision loss over 10 months (Figure S15). Prescribed with 900 mg/day idebenona for one year, she discontinued the drug due to diarrhea and chest pain. The patient had non-insulin-dependent diabetes mellitus and a history of cancerous stomach ulcer with surgical intervention. BCVA was 20/800 in both eyes. ERG showed normal scotopic response and slightly altered photopic response in both eyes. Multifocal ERG was normal. VEP showed decreased wave amplitude in all records. Terminal visual field was observed in both eyes.

### Cellular, biochemical, and molecular-genetic studies

Total DNA extraction followed standard methods. Screening for the three more frequent LHON mutations was performed by polymerase chain reaction (PCR) / restriction fragment length polymorphism (RFLP) analysis, as previously reported [4]. The m.3734A > G mutation was also analyzed by PCR / RFLP, using primers forward hmtL3568 (5′-CGCTCTTCTACTATGAACCC-3′) and reverse hmtH4501 (5′-TGTGCCTGCAAAGATGGTAG-3′). The amplicon size is 972 base pairs (bp), and the PCR conditions are 95 °C, 5 min (95 °C, 45 s / 64 °C, 45 s / 72 °C, 2 min) for 35 cycles, and 72 °C, 5 min. The restriction enzyme SchI cuts the wildtype sequence into 595 and 377 bp fragments, while the mutant sequence is cut into 595, 195, and 182 bp fragments. The complete mtDNA was amplified by long-range PCR, and overlapping fragments were sequenced according to previously described protocols [4]. The mtDNA copy number was determined by the quantitative real-time PCR method, as described elsewhere [5]. The *DNAJC30* gene was amplified and sequenced following previously described procedures [6].

Whole exome sequencing (WES) was performed on eight relatives as part of the URD-Cat program. Sequencing was conducted at the CNAG-CRG facility using the KAPA_HyperExomePlusMT_43Mb (Roche) capture kit aiming at 90X coverage. Sequencing data were analyzed according to the project’s established pipeline [7], using URD-Cat GPAP, a customized version of the RD-Connect GPAP [8].

A molecular model of ND1 subunit (PDB 5XTD) was obtained with the RasMol2.6 program (http://www.rasmol.org). For this analysis, glutamate 143 was in silico mutated to glycine with Swiss-PdbViewer v4.1.0.

Cytoplasmic hybrids (cybrids) were generated and grown, and capsaicin treatment was performed following published protocols [9, 10]. Karyotyping and genetic fingerprints of the cybrids were conducted as previously reported [11].

Growth rate in glucose or galactose, adenosine triphosphate (ATP) and reactive oxygen species (ROS) levels, oxygen consumption, respiratory complex IV (CIV), and citrate synthase (CS) specific activities, quantity of oxidative phosphorylation (OXPHOS) subunits, and mitochondrial messenger ribonucleic acid (mRNA) levels were measured following published protocols [4, 12, 13]. Oxygen consumption analysis was conducted according to established protocols [14], utilizing the high-resolution oxygraph OROBOROS® (Oroboros Instrument, Innsbruck, Austria). In summary, cells were harvested, washed, counted, and resuspended at 1 × 10^6^ cells/ml in DMEM. To correct for non-electron transport chain (ETC) related oxygen consumption, inhibition of mitochondrial respiration by KCN was performed. Respiration was measured at 37 °C, with chamber volumes set at 2 ml. Data acquisition and analysis were executed using the DatLab software at 1-s intervals [15]. Mitochondrial ATP levels, normalized by the cell number, were quantified using the CellTiter-Glow Luminiscent Cell Viability Assay with slight adaptations from established procedures [16]. Briefly, 10 000 cells/well were seeded 14 – 16 h before measurement. Cells were washed twice with PBS and incubated for 2 h in a record solution containing 5 mM 2-deoxy-D-glucose plus 1 mM pyruvate (oxidative ATP production). Cells were lysed, and lysates were incubated with the luciferin/luciferase reagents. Samples were measured using a microplate luminometer, with results normalized to cell count. Quantification of ROS production was performed as previously described [17, 18], with minor adaptations. CS was measured in 96-well plates, using freeze-thawing treated total cell homogenate and a standard protocol [19]. Activity data were normalized based on total protein content. Microplate assays were performed in a NovoStar MBG Labtech microplate instrument, and all enzyme determinations were performed in triplicate in at least three independent experiments.

### Statistical analysis

Data for mean and standard deviation are presented. Mann–Whitney U and Fisher exact tests, along with two-tailed unpaired t-tests, were used to compare parameters. *P*-values lower than 0.05 were considered statistically significant.

## Results

### DNA analysis

**Fig. 2:**
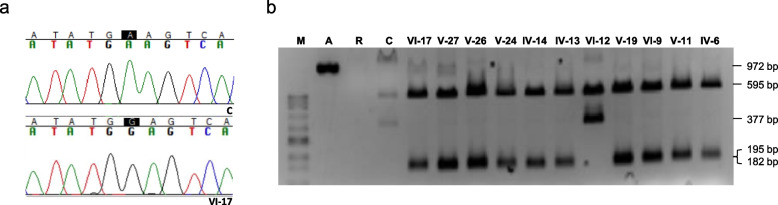
m.3734A > G genetic variant. **a** Electropherograms from a segment of control (C) and index (VI-17) mitochondrial deoxyribonucleic acid (mtDNA) sequences. **b** Picture of a gel showing the pattern of restriction fragment length polymorphisms. M, molecular weight marker; A, amplicon; R, rho^0^ (no mtDNA) sample; **C**, negative control

In light of the high occurrence of blindness among maternally-related family members (Fig. 1 and Table S1), a mother opted to have her healthy 6-year-old son (VI-17) tested for mtDNA because of maternal transmission of mtDNA (Figure S16). The analysis revealed an m.3734A > G transition in the *MT-ND1* gene (Fig. 2A). The affected maternal uncle (V-26) exhibited polymorphisms defining mtDNA haplogroup U6a1a1 (www.phylotree.org), including the variant, m.4172 T > A (*MT-ND1*)/p.L289Q, that affects an amino acid position already implicated in several other LHON cases, m.4171C > A/p.L289M [20–26]. Additionally, this individual had five apparently homoplasmic private genetic variants (GenBank: OQ803247): m.789 T > C in *MT-RNR1*, m.3734A > G in *MT-ND1*, m.9300G > A in *MT-CO3*, m.16183A > C, and m.16362 T > C in *MT-7SDNA* (Table S2). Among these, only m.3734A > G was a rare mutation [27]. The m.3734A > G transition has been previously reported only twice (haplogroups L2 and T) in more than 255 372 mtDNA complete sequences (www.mitomap.org), and has been recently suggested as the causative mutation of LHON in a Canadian patient [28]. Therefore, this mutation has appeared three times in individuals unrelated by descent from a common ancestor.

We confirmed the presence of this apparently homoplasmic mutation in the blood of 18 affected and 40 unaffected maternally related individuals in this pedigree (Fig. 2B). Pedigree analysis strongly supports maternal transmission of the phenotype, with 30 out of 95 maternally related offspring experiencing vision impairment, compared to 2 out of 104 maternally unrelated individuals (p < 0.00001, Fisher exact test). Moreover, two blind married couples, whose husbands were related through the maternal line and whose wives were non-LHON (IV-12, V-13), had non-blind offspring (Fig. 1).

To exclude nuclear genetic variants as the etiologic factor for this phenotype, we initially sequenced the *DNAJC30* gene, known to harbor LHON-causing mutations [29]. However, none of the variants proved to be the cause of the pathology (Table S3). Subsequently, WES was conducted on five affected individuals (IV-13, V-19, V-26, V-34, VI-25) and three unaffected individuals (V-24, VI-9, VI-30) who were maternally related in the pedigree (Fig. 1). No pathogenic variant associated with the clinical phenotype was detected. WES results revealed that three individuals were 100% mutant for the m.3734A > G transition, while the remaining five exhibited a mutation percentage greater than 98.1%.

### Protein analysis

**Fig. 3:**
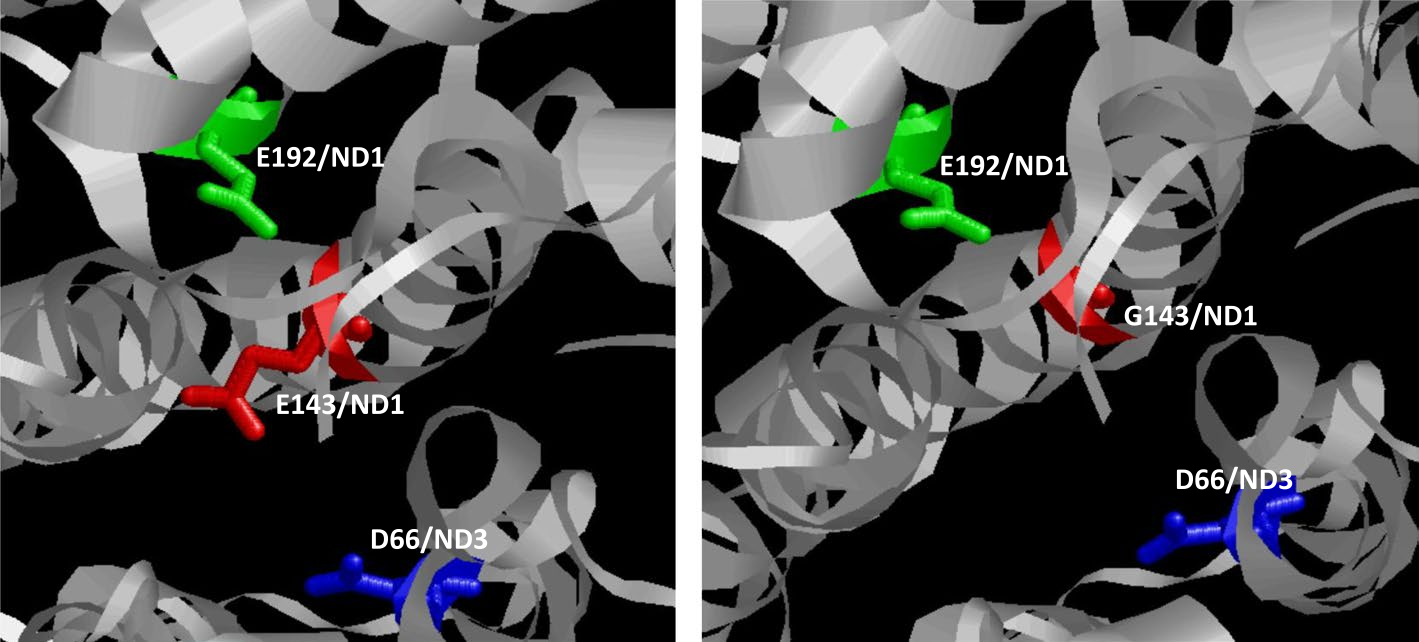
Molecular model of the protein environment of Glutamate 143 in ND1. Three acid amino acids involved in proton wire are remarked. D, aspartate; E, glutamate; G, glycine. ND1 and ND3, mitochondrial DNA-encoded respiratory complex I subunits 1 and 3, respectively

The m.3734A > G mutation induces a substitution of glutamate (E) with glycine (G) at position 143 (E143G) of the CI subunit ND1. E143 is conserved in 99.7% of 5165 ND1 sequences from Protists to Mammals [30]. Remarkably, two LHON-associated mutations at positions m.3733G > A and m.3733G > C of *MT-ND1* convert E143 to lysine (K) (E143K) and glutamine (Q) (E143Q) [22, 31]. The E143G substitution represents a non-conservative change, as glutamate is a large, polar, and negatively charged amino acid, whereas glycine is a small, non-polar amino acid. Notably, among the 52 possible glutamate-to-glycine substitutions in mtDNA-encoded CI subunits, only one has been reported more than 3 times (www.mitomap.org), underscoring the deleterious nature of this alteration. The pathogenicity predictor APOGEE (www.mitomap.org) designates this mutation as possibly pathogenic. Within the CI E-channel, a series of glutamates are deemed crucial for connecting ubiquinone reduction to proton translocation. Water molecules may facilitate proton transfer directly to E192/ND1. A Grotthuss-competent pathway for proton transfer exists within the E-channel, involving E192-S109/Y142-E143. E143/ND1 forms a water wire to D66/ND3 [32, 33]. However, the mutated residue, glycine, is not a Grotthuss-competent amino acid [34], thereby affecting this protons wire (Fig. 3).

### Functional assays

**Fig. 4:**
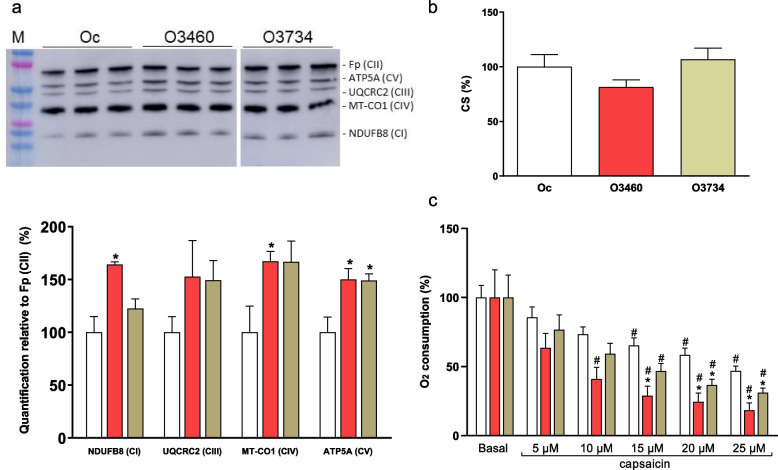
Phenotypic results from the study of negative control (Oc, white), positive control (O3460, red), and mutant (O3734, green) cybrids. **a** Levels of different protein subunits from oxidative phosphorylation (OXPHOS) complexes (in brackets) (*N* = 3). Picture of a Western blot immunodetection and quantitative graphic. M, molecular weight marker. **b** Citrate synthase specific activities (*N* = 3). **c** Oxygen consumption in cybrids exposed to growing concentrations of capsaicin. Means and standard deviations are shown (*N* = 4). Hashtags (#) and asterisks (*) indicate statistically significant differences (*p* < 0.050) versus the same cybrid without capsaicin and the negative control cybrid (Oc) at the same capsaicin concentration, respectively

To assess the pathogenicity of the m.3734A > G genetic variant, we conducted functional studies. Osteosarcoma 143B cybrids were generated from a negative control without any pathologic mutation (Genbank: OQ803246, Oc), a positive control containing the m.3460G > A LHON pathologic mutation in *MT-ND1* (GenBank: JX401416, O3460), and the patient (GenBank: OQ803247, O3734). Through karyotyping and genetic fingerprinting, we verified that these cybrids shared the same nDNA genetic background as the osteosarcoma 143B rho^0^ cell line. The presence or absence of the m.3734A > G mutation was also confirmed. mtDNA levels exhibited no significant differences among cybrids (Figure S17). Various parameters, including glucose/galactose growth ratio, oxygen consumption, ROS production, ATP amount, and mRNA levels, were examined and revealed no significant differences among cybrids (Figure S18). Although CS specific activity, a marker of the cell fraction occupied by mitochondria, remained unchanged, the protein levels of different OXPHOS complexes tended to increase (the increase in CV ATP5A subunit was statistically significant) (Fig. 4A,B). These results suggest that OXPHOS biogenesis could compensate for the previously determined parameters. Interestingly, our prior observations indicated that cybrids with the m.3460G > A LHON mutation in ND1 were more sensitive to capsaicin than control cybrids or those with the other classical mutations in ND4 and ND6 [10]. Similarly, the O3734 cybrid displayed increased sensitive to capsaicin compared to the Oc (Fig. 4C).

### Pedigree analysis

This pedigree spans seven generations, comprising a total of 199 individuals. The disease expression shows a decreasing penetrance along generations, with rates of 66.7% (2nd), 45.5% (3th), 36.8% (4th), 39.3% (5th) and 18.5% (6th). The 1st generation serves as the founder ancestor, and 7th generation is excluded from analysis due to the youth of its representatives. The age at onset ranges from 4 to 78 years, with a mean onset age of 29 years. Earlier generations, being older, exhibited a higher penetrance for blindness.

A significant excess of affected males (15/31 – 48.4%) compared to affected females (15/64—23.4%) is observed (*p* = 0.019, Fisher exact test). The age of onset is significantly lower in males [18.0 yo ± 6.3 (15)] than in females [41.8 yo ± 22.4 (13)] (*p* = 0.0005, unpaired t test). Although there is a higher proportion of affected smokers (14/33 – 42.4%) than affected non-smokers (12/44 – 27.3%), this difference does not reach statistical significance. The age of onset is not significantly lower in smokers [25.3 yo ± 20.2 (16)] than in non-smokers [27.6 yo ± 13.9 (14)].

Examining the pedigree reveals two main branches with different disease penetrance. The branch starting from III-1 includes 13 (61.9%) affected individuals out of 21 maternally related individuals. The other branch, starting from III-3, incorporates 9 (19.6%) affected individuals out of 46 maternally related individuals. Environmental factors or alleles in nuclear genes could potentially explain this difference [35]. However, it is most likely attributed to the variation in the number of males, who are more susceptible, in each branch. The first branch has 12 (57.1%) males, a statistically significant excess (*p* = 0.0016, Fisher exact test) compared to the second branch, which has 8 (17.4%) males. This trend is also observed in the smaller branches. Thus, the penetrance and percentage of males in branch II-3 are 40.0% and 50.0%, respectively, while in branch III-7, they are 22.2% and 22.2%, and in branch III-9, 0% and 25%, respectively. In addition, the penetrance among males in the branch starting in III-1 is higher (9/12, 75%) than in the branch starting in III-3 (3/8, 37.5%). Similar trends are observed among females, with higher penetrance (4/9, 44.4% versus 6/38, 15.8%) and significantly higher ages (*P* = 0.0343, unpaired t-test) in branch III-1 females [52.5 ± 19.7 (6)] compared to branch III-3 females [33.8 ± 19.3 (36)]. Similar but nonsignificant differences are observed in males [43.2 ± 20.2 (9) versus 31.5 ± 26.8 (8)]. Finally, there are no significant differences in the frequency of smoking between these two branches of the family.

## Discussion

In this study, we present and functionally characterize a novel pathological mutation within a sizable LHON family.

Even the three most common LHON variants have a relatively minor effect on enzymatic activity. LHON is characterized by the selective degeneration of retinal ganglion cells (RGCs), which are the terminal retinal neurons projecting their optic nerve-forming axons to the brain, leading to permanent blindness. Despite the presence of causative mutations in all tissues, only a specific cell type is affected, suggesting dependence on tissue and nuclear genetic background. Thus, it has been suggested that “routinely used functional studies of respiratory chain activities may not be helpful in verifying the pathogenicity of LHON variants” [36], and “There is much evidence that even confirmed pathogenic LHON mutations may not display any detectable respiratory chain defect” [37]. The lack of suitable models further hinders our understanding of the underlying pathogenic mechanism [38]. Despite functional assays showing no significant differences, multiple pathogenicity criteria, including population frequency, mutations in the same amino acid associated with LHON, non-conservative substitution, high amino acid conservation index, participation in the proton wire pathway, and pedigree results, collectively support the pathogenicity of the mutation m.3734A > G.

Similar to many LHON mutations, the m.3734A > G mutation exhibits incomplete penetrance and significant sex and age dependence for the onset of clinical manifestations. The decrease in penetrance across new generations reflects the younger age of individuals in these generations, implying lower penetrance if age is considered a risk factor. Older age generally implies a greater possibility of being exposed to more risk factors. The penetrance of the mutation in the presented pedigree (31.6%) surpasses that of another large published LHON pedigree (24.8%) [3]. Curiously, the percentage of males in the pedigree with the m.3734A > G mutation (32.6%) is considerably lower than that observed in this m.11778G > A family (51.2%). It is plausible that the pathological mutation, a nuclear genetic factor, or the mtDNA haplotype may contributed to the elevated penetrance observed in our pedigree. Interestingly, the presence of mutations at two amino acid positions associated with LHON, L289 and E143, both affecting the ND1 subunit of CI, might contribute to the heightened penetrance despite a lower percentage of males. Comparative analyses with population studies suggest that the high penetrance in this pedigree is potentially influenced by additional modifying factors [39]. Therefore, this extensive family offers a valuable opportunity to explore other factors, whether genetic or environmental, that may modulate penetrance.

### Supplementary Information

**Supplementary Material 1.**

## Data Availability

The datasets used and/or analyzed during the current study are available from the corresponding author on reasonable request. The data are not publicly available due to privacy and ethical restrictions.
