# Supplementary material for: Identification and characterization of a new pathologic mutation in a large Leber hereditary optic neuropathy pedigree
Source: Orphanet J Rare Dis. 2024 Apr 6;19:148. doi: 10.1186/s13023-024-03165-2 (PMC10999093; doi:10.1186/s13023-024-03165-2)
Supplement: Supplementary file 1 — Supplementary Material 1. [file 13023_2024_3165_MOESM1_ESM.docx]

**Supplementary Information**

Supplementary Figures (Figure S1 - Figure S18)

Supplementary Tables (Table S1 – Table S3)

**Supplementary Figures**

**Figure S1.** A slight thickening of the retinal nerve fibre layer (RNFL) was found in the right eye of patient 1 in August 2018.

**Figure S2.** Central scotoma was present in both eyes in November 2018, although it was much larger in left eye.


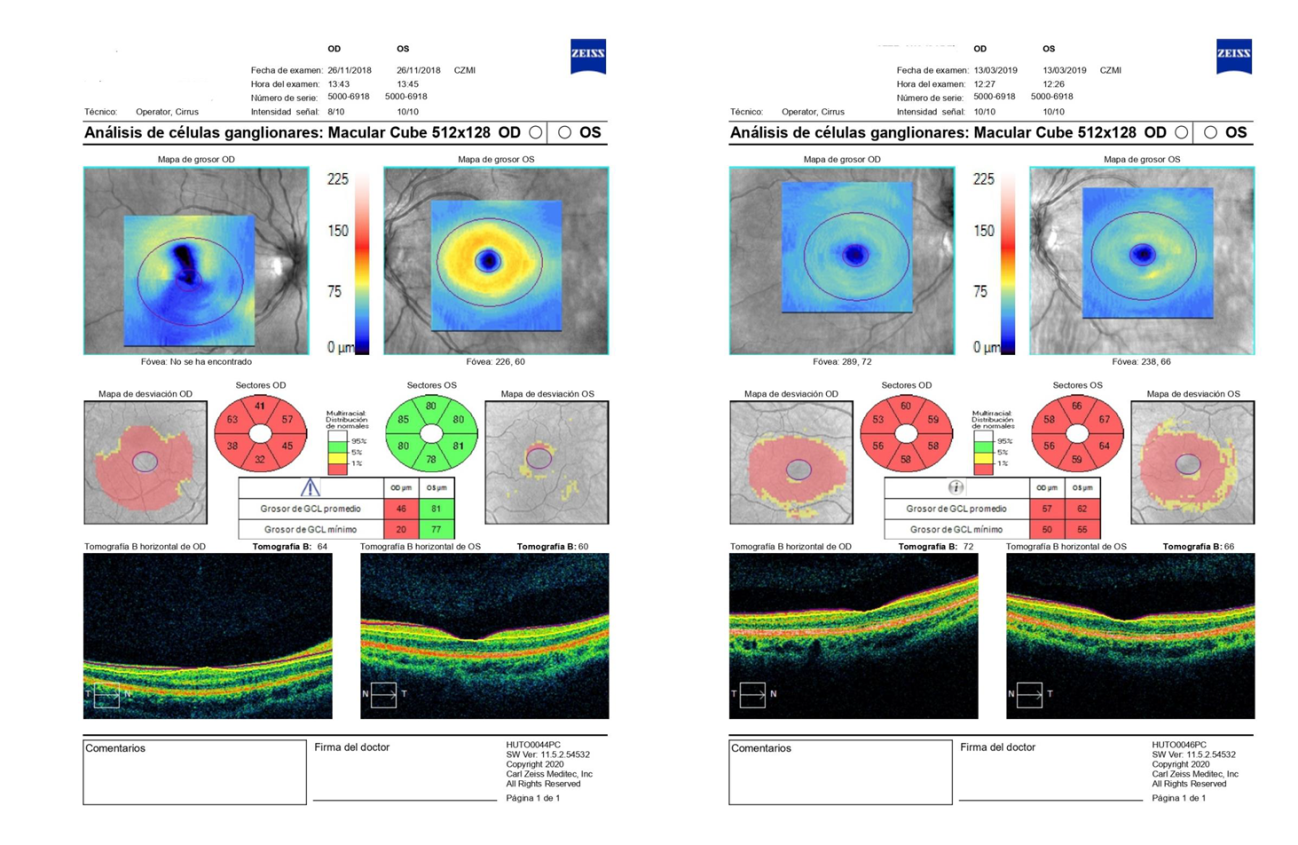


**Figure S3.** Evolution of ganglion cell layer (GCL) thickness in both eyes from November 2018 to march 2019.

**Figure S4.** Both eyes of patient 2 suffered from central scotoma in July 2020.


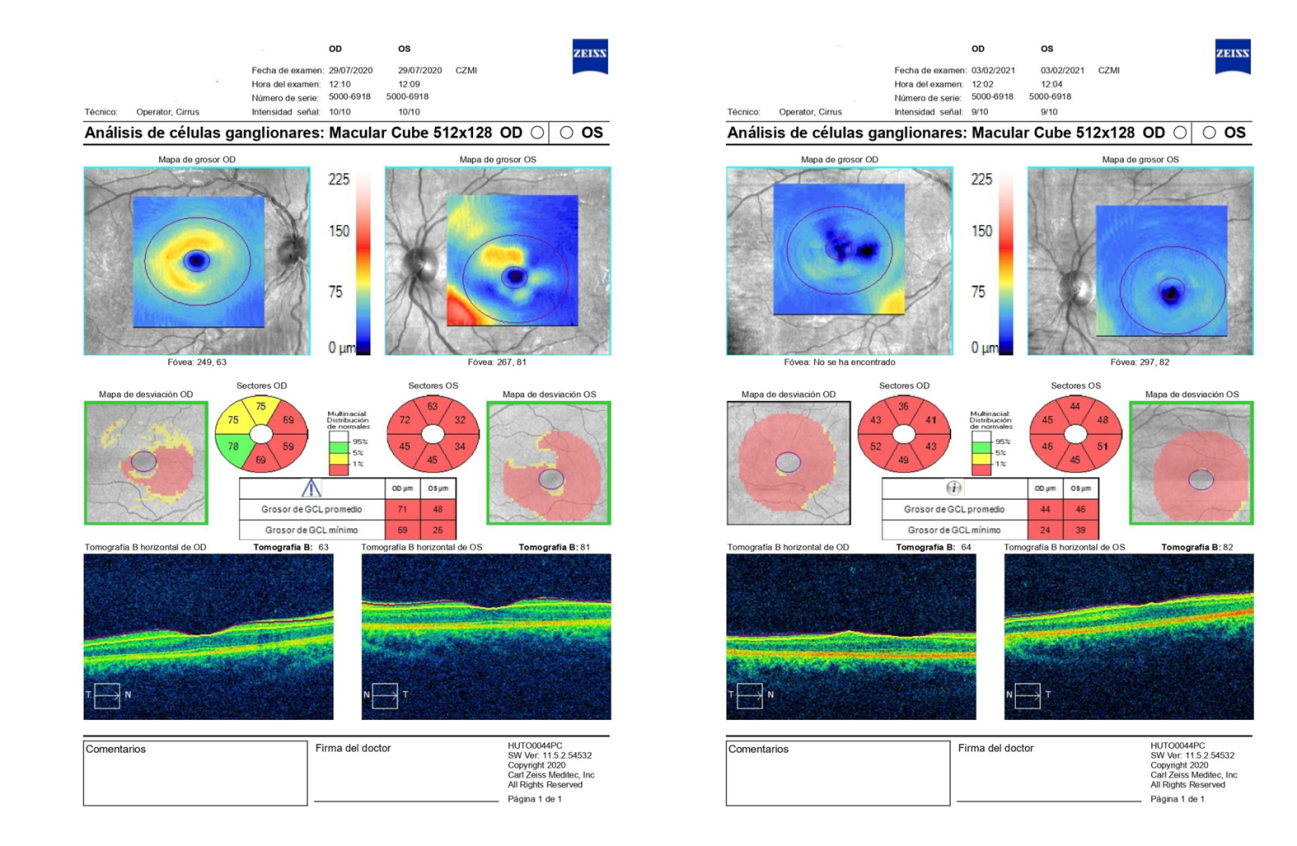


**Figure S5.** Ganglion cell layer (GCL) evolved to severe atrophy in both eyes within a few months.

**Figure S6.** After a 2-year follow-up, both eyes showed a severe thinning of the retinal nerve fibre layer (RNFL).

**Figure S7.** In just one month, visual field was abolished in right eye and a large central scotoma developed in left eye.


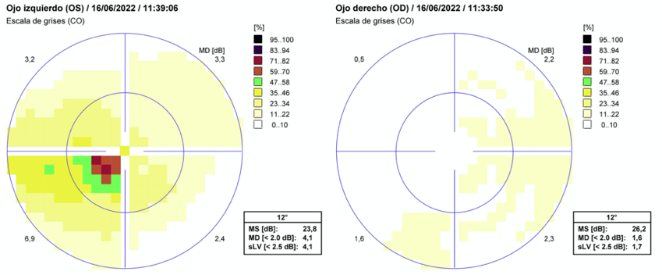

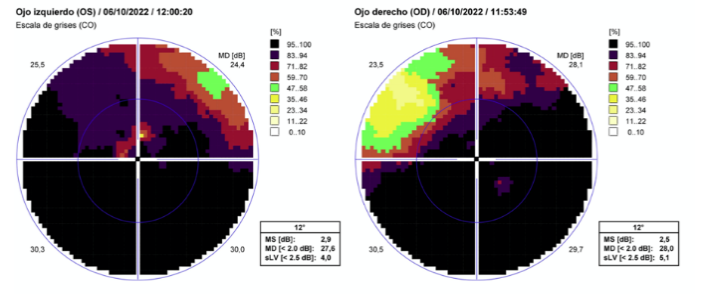


**Figure S8.** Octopus visual field (30-2) made in June 2022 (left) where left eye showed a centrocecal campimetric defect. Right eye showed no defects. Octopus visual field made in October 2022 (right) revealed a clear worsening, presenting absolute scotomas in both eyes.


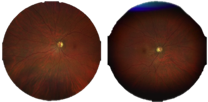


**Figure S9.** Retinography of the right (on the left) and left (on the right) eye. Selective temporal atrophy is more evident in the left eye.


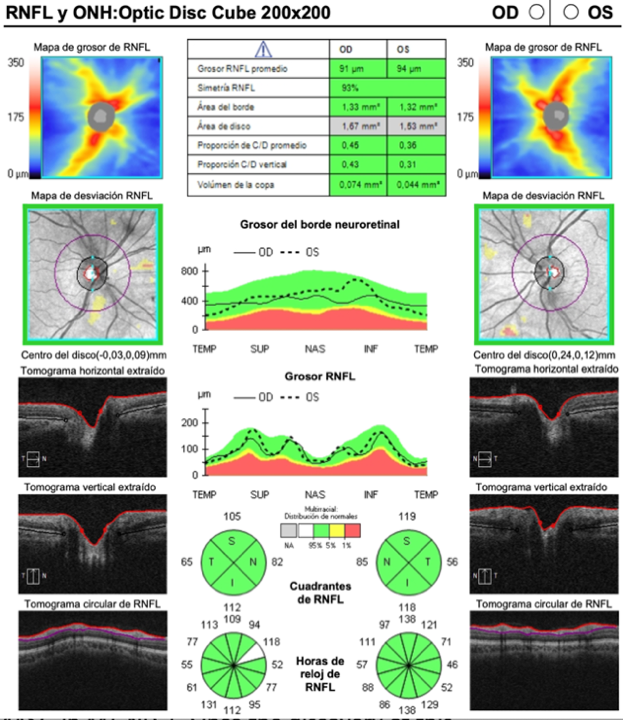

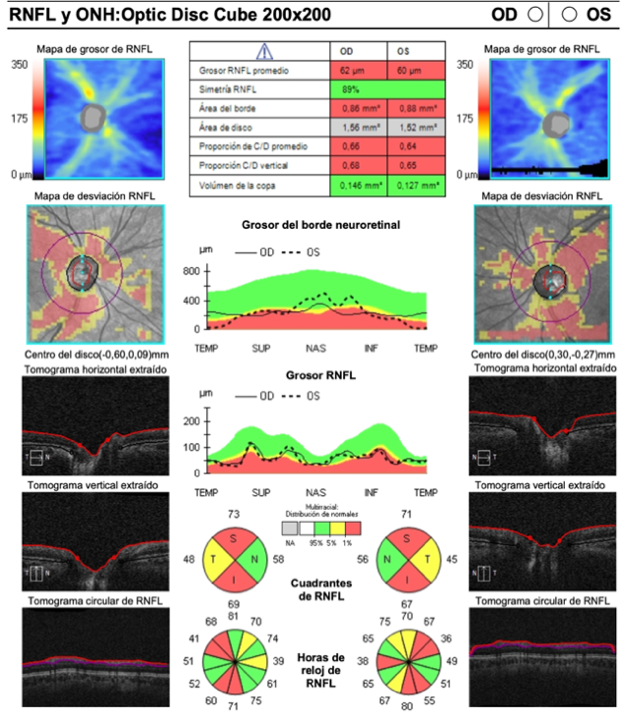

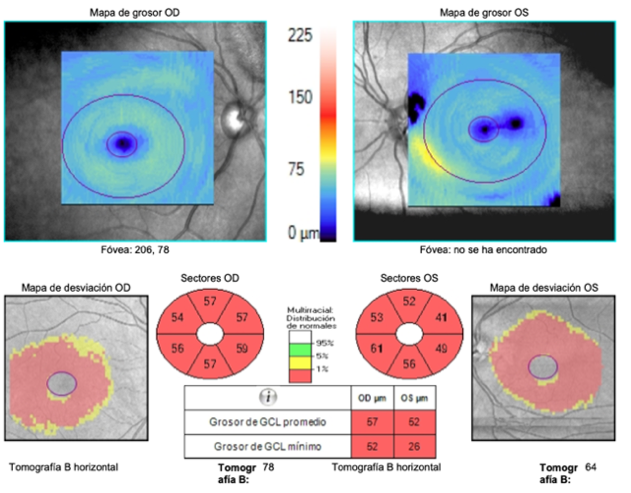


**Figure S10.** Peripapillary retinal nerve fiber layer (RNFL) thickness measured by optical coherence tomography (OCT) scan in June 2022 (left) and in June 2023 (right) revealed a worsening of the retinal health of the patient in one year, with thinning in superior and inferior sectors in both eyes. This thinning is also seen in the ganglion cell density of both eyes.

*
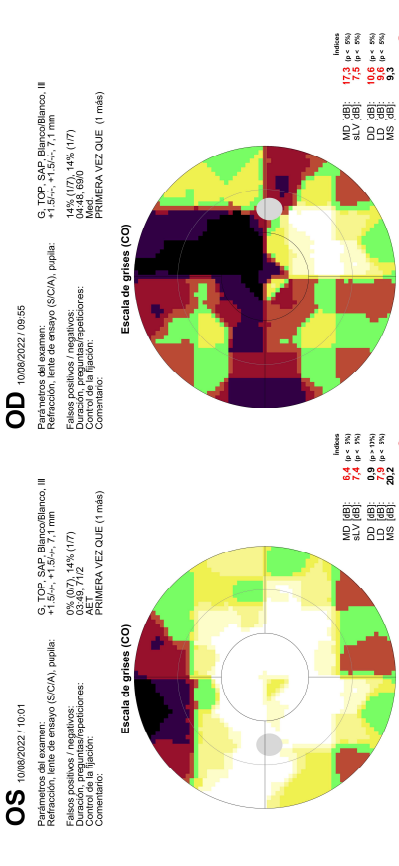

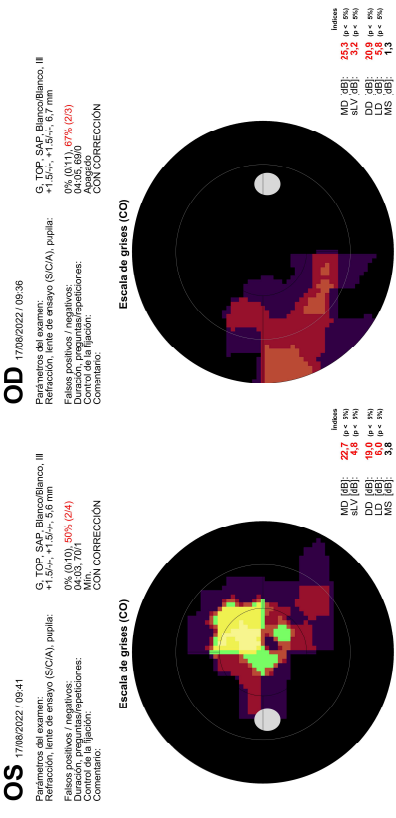
*

**Figure S11.** Octopus visual field (30-2) made in August 2022 (left) where right eye showed a large scotoma. One week later (visual field on the right), a clear worsening is seen. Indeed, the visual field revealed a minimal central island of vision in both eyes.


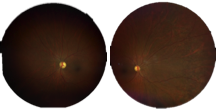


**Figure S12.** Retinography of the left (on the left) and right (on the right) eye. Selective temporal atrophy is more evident in the left eye.


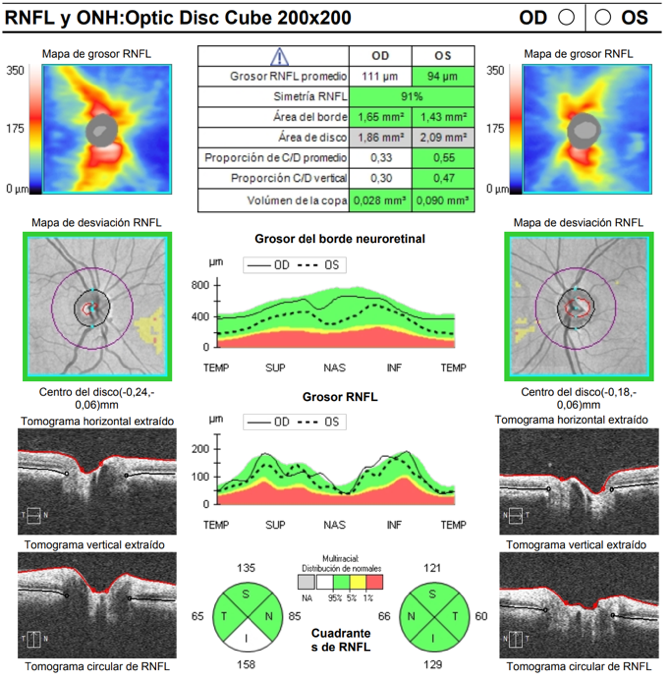

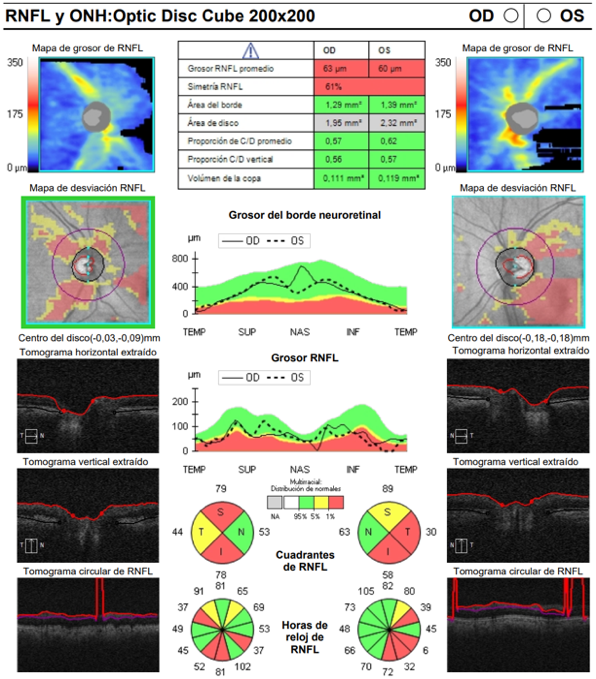

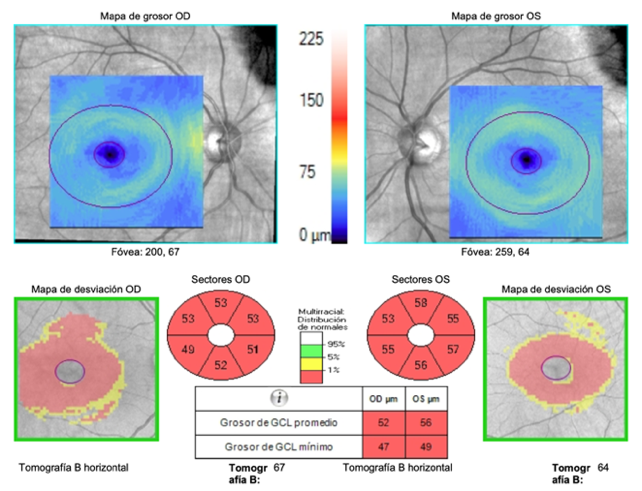


**Figure S13.** Peripapillary retinal nerve fiber layer (RNFL) thickness measured by optical coherence tomography (OCT) scan in August 2022 (left) and in May 2023 (right) revealed a worsening of the retinal health of the patient in months, with thinning in superior and inferior sectors in the right eye and thinning on the temporal and inferior sectors in the left eye. This thinning is also seen in the ganglion cell density of both eyes.

**Figure S14.** Patient 6. a) Optical coherence tomography (OCT) shows retinal nerve fiber layer (RNFL) thinning of all the quadrants in both eyes, including optic nerve atrophy. b) Ganglion cell layer (GCL) was affected, mainly in the left eye (OS), with optic atrophy. Thinned macula in both eyes. c) Automated Humphrey visual field using the Humphrey field analyzer and 30-2 algorithm showed loss of vision, with scotoma in OS and central scotoma and relative peripheral in OS. d) Dark- and light-adapted electroretinogram according to International Society for Clinical Electrophysiology of Vision standards showed normal scotopic and photopic amplitudes. e) Right and left fundus autofluorescence photographs. f) Right and left retinography. OD, right eye; VFI, visual field index.

**Figure S15.** Patient 7. a) Optical coherence tomography (OCT) shows retinal nerve fiber layer (RNFL) thinning of the temporal quadrant with thickening of all the other quadrants in both eyes, including optic nerve atrophy. b) Ganglion cell layer (GCL) was affected in both eyes. Macula slightly thinned in both eyes. c) Automated Humphrey visual field using the Humphrey field analyzer and 30-2 algorithm showed loss of vision. d) Dark- and light-adapted electroretinogram according to International Society for Clinical Electrophysiology of Vision standards showed normal scotopic and decreased amplitude in photopic. e) Right and left fundus autofluorescence photographs. f) Right and left retinography. OD, right eye; OS, left eye; VFI, visual field index.

**Figure S16.** Analysis of the classical mutations, plus the m.14459G>A, for Leber hereditary optic neuropathy. M, molecular weight marker; A, amplicon; C, negative control; P, positive control.

**Figure S17.** Genetic characterization of negative control (Oc), positive control (O3460), and mutant (O3734) cybrids. a) Karyotypes. b) Genetic fingerprint. c) Gels showing the patterns of restriction fragment length polymorphisms for the m.3460G>A and m.3734A>G genetic variants. d) Electropherograms from a segment of Oc and O3734 mtDNA sequences. e) mtDNA copy number. Means and standard deviations are shown (N = 4).

**Figure S18.** Functional analysis of negative control (Oc, white), positive control (O3460, red), and mutant (O3734, green) cybrids. a) Proliferation rate in glycolytic (glucose) and oxidative (galactose) conditions (N = 3). b) Oxygen consumption (N = 4). c) Amount of reactive oxygen species (ROS) (N = 16). d) Mitochondrial ATP quantity (N = 20). e) Mitochondrial mRNA levels for different mtDNA-encoded genes (N = 3). Means and standard deviations are shown. Asterisks (*), p < 0.050 versus Oc.

**Supplementary Tables**

| **Tree position** | **Age** | **Sex** | **Vision lost** | **Age of**  **onset** | **Smoker** | **Mutation** |
| --- | --- | --- | --- | --- | --- | --- |
| I-1 | Death | F |  |  |  |  |
| II-1 | Death | F | Y |  | N |  |
| II-3 | Death | F | Y |  | N |  |
| II-6 | Death | M | N |  |  |  |
| III-1 | Death | F | Y | 29 | N |  |
| III-3 | Death | F | N |  | N |  |
| III-6 | Death | M | N |  |  |  |
| III-7 | Death | F | N |  | N |  |
| III-9 | 98 | F | N |  | N | Y |
| III-12 | Death | M | Y | 20 | Y |  |
| III-14 | Death | M | N |  |  |  |
| III-15 | Death | M | Y | 20 |  |  |
| III-16 | Death | F | N |  |  |  |
| III-17 | Death | F | Y | 55 |  |  |
| III-19 | Death | F | Y | 65 |  |  |
| IV-2 | Death | M | Y | 17 | Y |  |
| IV-3 | Death | M | Y | 18 | Y |  |
| IV-5 | Death | M | Y | 12 | N |  |
| IV-6 | 79 | F | N |  | N | Y |
| IV-8 | Death | F | N |  | N |  |
| IV-10 | Death | F | Y | 45 | N |  |
| IV-13 | 73 | M | Y | 20 | N | Y |
| IV-14 | 71 | F | Y | 71 | Y | Y |
| IV-16 | 80 | F | Y | 78 | Y | Y |
| IV-19 | 82 | M | N |  |  |  |
| IV-20 | 74 | F | N |  | N | Y |
| IV-24 | 74 | F | N |  | N | Y |
| IV-26 | 68 | F | N |  | N | Y |
| IV-27 | 65 | F | N |  | Y | Y |
| IV-29 | 71 | F | N |  | N | Y |
| IV-39 |  | M | N |  |  |  |
| IV-40 |  | F | N |  |  |  |
| IV-41 |  | F | N |  |  |  |
| IV-42 |  | M | N |  |  |  |
| V-11 | 45 | F | Y | 40 | Y | Y |
| V-14 | 57 | M | Y | 20 | Y | Y |
| V-15 | 52 | F | N |  | Y | Y |
| V-17 | 45 | M | Y | 16 | Y | Y |
| V-19 | 50 | M | Y | 13 | Y | Y |
| V-20 | 49 | M | Y | 14 | Y |  |
| V-24 | 48 | M | N |  | Y | Y |
| V-26 | 45 | M | Y | 14 | Y | Y |
| V-27 | 43 | F | N |  | Y | Y |
| V-29 | 59 | M | Y | 14 | Y | Y |
| V-30 | 58 | F | N |  | N | Y |
| V-32 | Death | F | N |  | Y |  |
| V-34 | 45 | F | Y | 4 | N | Y |
| V-39 | 58 | F | N |  | N | Y |
| V-41 | 57 | F | N |  | Y |  |
| V-42 | 55 | F | N |  | Y |  |
| V-44 | 53 | F | N |  | Y | Y |
| V-46 | 52 | F | N |  | Y | Y |
| V-48 | 50 | F | N |  | N | Y |
| V-50 | 48 | F | Y | 48 | Y | Y |
| V-52 | 44 | F | N |  | Y | Y |
| V-54 | 41 | F | N |  | N | Y |
| V-56 | 33 | F | N |  | N | Y |
| V-57 | 48 | F | Y | 48 | N | Y |
| V-59 | 43 | F | N |  | N | Y |
| V-61 | 38 | M | Y | 38 | N | Y |
| V-62 | 50 | M | N |  | N | Y |
| V-63 | 33 | F | N |  | N |  |
| VI-9 | 11 | M | N |  | N | Y |
| VI-11 | 25 | F | N |  |  |  |
| VI-17 | 11 | M | N |  | N | Y |
| VI-18 | 28 | F | Y | 20 | Y | Y |
| VI-20 | 22 | F | N |  | Y | Y |
| VI-21 | 35 | F | N |  | Y |  |
| VI-23 | 32 | F | N |  | Y | Y |
| VI-25 | 18 | M | Y | 14 | N | Y |
| VI-26 | 12 | F | N |  | N | Y |
| VI-30 | 36 | M | N |  | N | Y |
| VI-31 | 29 | F | N |  | N | Y |
| VI-32 | 27 | F | N |  | Y | Y |
| VI-33 | 34 | F | N |  | N | Y |
| VI-34 | 29 | F | N |  | Y |  |
| VI-35 | 27 | F | N |  | Y | Y |
| VI-36 | 17 | F | N |  | N | Y |
| VI-37 | 35 | F | N |  | N | Y |
| VI-39 | 29 | F | Y | 25 | N | Y |
| VI-40 | 23 | M | Y | 20 | N | Y |
| VI-41 | 19 | F | N |  |  | Y |
| VI-42 | 25 | F | N |  | Y | Y |
| VI-43 | 23 | M | N |  | Y | Y |
| VI-44 | 22 | F | N |  | Y | Y |
| VI-45 | 17 | F | Y | 15 | Y | Y |
| VI-46 | 6 | F | N |  | N | Y |
| VI-47 | 18 | F | N |  | N |  |
| VI-48 | 10 | M | N |  | N | Y |
| VII-4 | 8 | F | N |  | N | Y |
| VII-5 | 2 | M | N |  | N |  |
| VII-6 | 9 | M | N |  | N |  |
| VII-7 | 5 | F | N |  | N |  |
| VII-8 | 9 | F | N |  | N | Y |
| VII-9 | 2 | F | N |  | N |  |

**Table S1**. Demographic and clinical variables of maternally related individuals from this pedigree. Individuals affected of visual lost are marked in red color.

mtDNA- revised Cambridge reference sequence (GenBank, NC_012920)

m.789T>C dbSNP (rs879211540) *MT-RNR1* (HGNC:7470)

m.3734A>G dbSNP (rs1603219106) *MT-ND1* (HGNC:7455)

m.9300G>A dbSNP (rs371745772) *MT-CO3* (HGNC:7422)

m.16183A>C dbSNP (rs28671493) *MT-7SDNA* (HGNC:7409)

m.16362T>C dbSNP (rs62581341) *MT-7SDNA* (HGNC 7409)

*DNAJC30* gene (GenBank, NM_032317.3)

c.34C>G dbSNP (rs28494095) *DNAJC30* (HGNC:16410)

c.100G>A dbSNP (rs1128349) *DNAJC30* (HGNC:16410)

c.516C>T dbSNP (rs1569062) *DNAJC30* (HGNC:16410)

**Table S2**. Genes and sequence variants. GenBank, https://www.ncbi.nlm.nih.gov/genbank/; dbSNP, https://www.ncbi.nlm.nih.gov/snp/; HGNC, https://www.genenames.org/.

**Table S3.** Genetic variants in the coding region of the *DNAJC30* gene. (Reference sequence: NM_032317.3)
